# A case report of iatrogenic gas gangrene post colonoscopy successfully treated with conservative management- is surgery always necessary?

**DOI:** 10.1186/s12876-020-01314-y

**Published:** 2020-05-27

**Authors:** Lachlan J. Fairley, Samuel L. Smith, Stephen K. Fairley

**Affiliations:** 1 College of Medicine and Dentistry, James Cook University, Townsville, Australia; 2 Princess Alexandra Hospital, Woolloongabba, Brisbane, Australia; 3 Queensland Research Centre for Peripheral Vascular Disease, College of Medicine and Dentistry, Townsville, Australia; 4 Royal Brisbane and Women’s Hospital, Herston, Brisbane, Australia; 5 The Mater Hospital, Townsville, Australia

**Keywords:** Colonoscopy, Clostridium infections, Gas gangrene, Intestinal disease, Conservative treatment, Treatment outcomes, Postoperative complications, Case report

## Abstract

**Background:**

Colonoscopy is a routine procedure in diagnosis and treatment of colonic disease. While generally regarded as a safe procedure, potentially fatal complications can occur. Gas gangrene is one such complication, with very high mortality. There are few cases of gas gangrene occurring after colonoscopy, making it one of the rarer complications of this procedure. There have been no previously reported cases of a patient surviving such an infection and the optimal treatment strategy is contentious. This report describes a case of intramural gas gangrene of the colon, treated conservatively with antibiotic therapy in which the patient survived with full recovery.

**Case presentation:**

A 71-year-old, previously healthy male presented 6 h post apparently uncomplicated colonoscopic polypectomy with rigors, nausea, vomiting and right upper quadrant pain. At presentation he was febrile at 40.1 °C but hemodynamically stable. Abdominal computed tomography revealed substantial colonic thickening and several focal intramural gas bubbles (pneumatosis intestinalis) surrounding the polypectomy site. Within 24 h post procedure he became hypotensive and was admitted to ICU in frank septic shock requiring inotropes, and with demonstrable septic myocardial depression. Bloods showed multi-organ derangement with leukocytosis, lactic acidosis, haemolytic anaemia and hyperbilirubinemia. A diagnosis of presumed Clostridial gas gangrene was made, and treatment was initiated with benzylpenicillin, clindamycin, metronidazole and vancomycin. After 4 days in ICU he was stepped down, and discharged after a further 10 days with no surgical or endoscopic interventions. At three-month review he reported being back to full health.

**Conclusions:**

This case demonstrates that gas gangrene infection is a possible complication of colonoscopic polypectomy. This is a cause of rapid deterioration in post-colonoscopy patients and has been misdiagnosed as colonic perforation in previously reported cases of retroperitoneal gas gangrene. Such misdiagnosis delays antibiotic therapy, which likely plays a role in the high mortality of this condition. Early diagnosis and initiation of antibiotic therapy with benzylpenicillin and clindamycin as seen in this case is essential for patient survival. While surgery is typically performed, non-operative management of pneumatosis intestinalis, and potentially gas gangrene is becoming more common and was utilized effectively in this patient.

## Background

Colonoscopy is a routine procedure in the screening, diagnosis and treatment of colonic disease. Reports estimate that more than 800,000 colonoscopies are performed each year in Australia, and this number is increasing [1]. Beyond simple visualisation of the colon, colonoscopy can also be used for interventions such as polypectomy. Both colonoscopy and polypectomy are generally regarded as safe procedures, although complications may occur [2]. Whilst most complications are generally harmless, there is the potential for more serious complications to occur, such as colonic perforation, which has been reported to occur in almost 1 in 1000 procedures [2]. Perforation most commonly occurs from instrumentation, however infection is a recognised, albeit rare, cause of perforation post-colonoscopy. Another related recognised complication is bacteraemia, which may arise from a patient’s own microbial flora or from microbes introduced endoscopically, and is reported to occur in 2.2% of all colonoscopies [3]. This bacteraemia however is usually transient and asymptomatic, even with isolates of known pathogenic bacteria [3]. Gas gangrene is a frequently fatal condition characterised by tissue necrosis with the formation of gas bubbles, most commonly secondary to infection by *Clostridium spp*. [4] Whilst it is a rare complication in the setting of colonoscopy, it can be rapidly fatal as a result of perforation or septicaemia [5, 6]. A literature review identified only 3 cases of abdominal gas gangrene following colonoscopy, all of which were treated surgically and were universally fatal [7–9]. Below we describe a case of colonic gas gangrene occurring shortly after colonoscopy in which the patient survived with non-surgical management with excellent recovery.

## Case presentation

We report the case of a 71-year-old, previously healthy male, who was admitted through the emergency department of a North Queensland hospital with rigors, right upper quadrant pain, nausea and vomiting, 6 h after an apparently uncomplicated colonoscopic polypectomy. The colonoscopy was performed as part of routine bowel cancer screening following a positive faecal occult blood test. During the procedure, a single large polyp (15 mm × 31 mm) was removed from the proximal transverse colon with submucosal adrenaline injection and diathermy. Visual inspection at the time showed a clean base with no suggestion of perforation.

At the time of his emergency admission he was febrile at 40.1 °C, heart rate was 102 beats/min and regular, blood pressure was 131/68 mmHg, his respiratory rate was 20 breaths/minute and SpO2 was 98% on room air. Physical examination revealed a soft abdomen with right upper quadrant tenderness. The remainder of his cardiorespiratory examination was unremarkable. Initial bloods revealed leukocytosis with left shift and mildly elevated C-reactive protein (CRP), ALP and GGT, but normal electrolytes and renal function. Blood cultures were taken prior to initiation of antibiotics, which failed to grow any organisms. Chest X-ray showed no evidence of subdiaphragmatic gas or other infective processes. The patient was admitted and initiated on intravenous (IV) piperacillin-tazobactam.

**Fig. 1:**
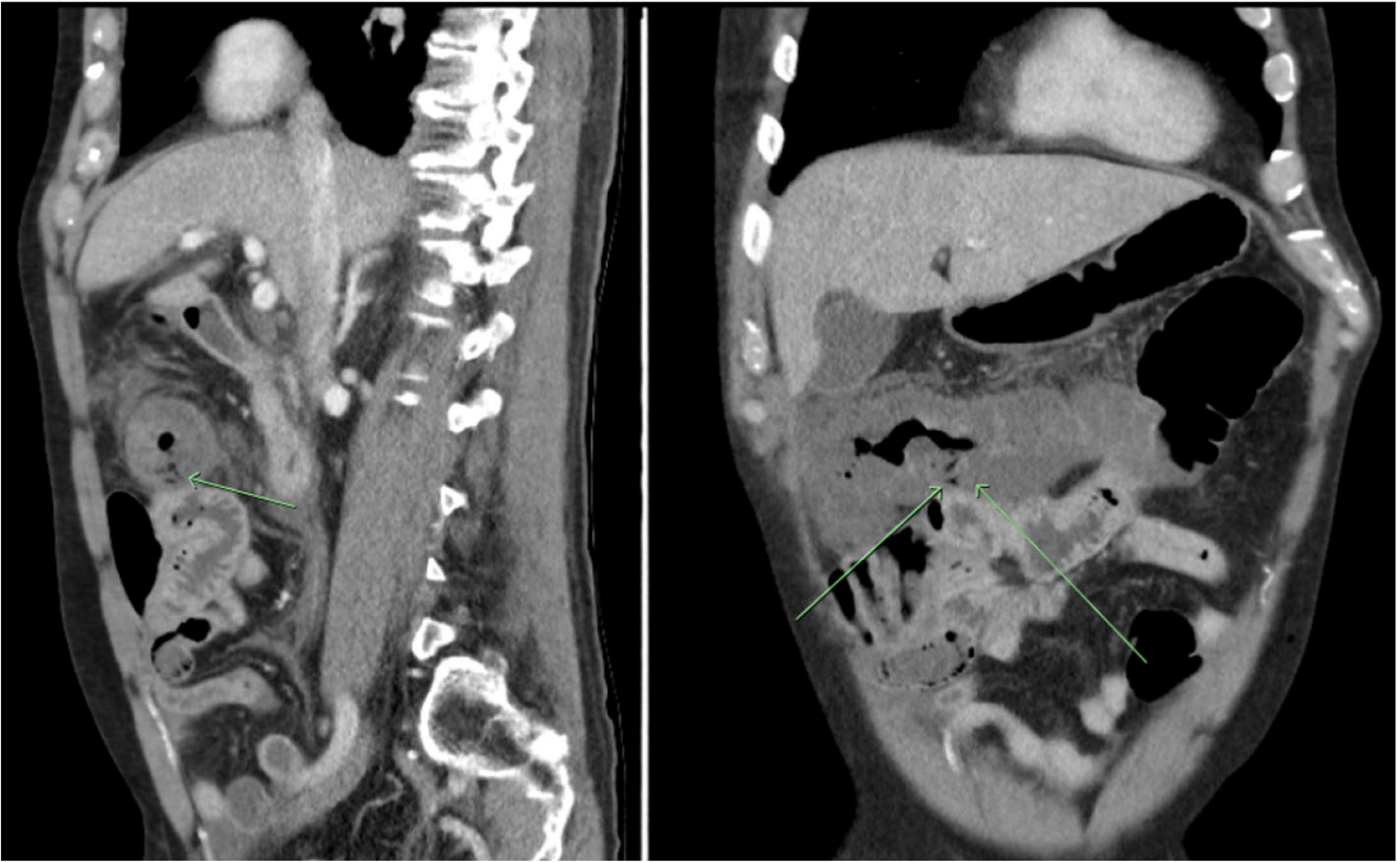
Abdominal CT, sagittal and coronal views, showing mural thickening of the proximal transverse colon and hepatic flexure with arrows highlighting visible intramural gas

A CT scan the following morning revealed substantial thickening of the bowel wall of the hepatic flexure and proximal transverse colon with induration of surrounding mesocolic fat, as well as several focal intramural gas bubbles localised to the polypectomy site (Fig. 1). There was no evidence of pericolic gas or pneumoperitoneum. Soon afterwards this same day, the patient became hypotensive, with a systolic blood pressure of < 60 mmHg despite 3 L of IV fluid resuscitation. He was transferred to the intensive care unit (ICU) with rapidly deteriorating vital signs, becoming hypotensive tachycardic and hypoxic. Arterial blood gas analysis showed a pH of 7.23, HCO_3_^−^ of 12.1 mmol/L and a serum lactate of 3.8 mmol/L rising to 4.8 mmol/L on serial analysis. Once in ICU he received a further 1 L of Hartman’s solution and 200 mL of albumin. Bedside echocardiography showed moderate left ventricular systolic dysfunction consistent with septic myocardial depression. The patient was given inotropic support with dobutamine and noradrenaline infusions, however he did not require intubation.

A diagnosis of clostridial myonecrosis (gas gangrene) was made, supported by the rapid deterioration of the patient, demonstrable intramural gas on CT, raised lactate potentially indicative of gut hypoperfusion and the inciting event of recent colonoscopy. Further investigations in ICU revealed a new haemolytic anaemia, with a fall in haemoglobin from 163 g/L to 120 g/L, with raised total bilirubin (45 micromol/L) and lactate dehydrogenase (371 U/L). This was consistent with the intravascular haemolysis sometimes seen with clostridial infections.

The patient’s IV antibiotics were changed to clindamycin 900 mg 8 hourly and benzylpenicillin 2.4 g 6 hourly to cover for *Clostridium*, in addition to meropenem 1 g 8 hourly and vancomycin 250 mg 6 hourly for other potential causes of sepsis. Colorectal surgery was consulted for potential surgical management with resection, however a trial of medical management with IV antibiotics was decided upon.

The evening of his ICU admission there was one episode of melaena. The patient remained unstable requiring IV fluids and inotropic support for 2 days before his cardiovascular stability and acidosis began to improve, and he was able to be weaned from inotropic support. He was stepped down from ICU to the ward after 4 days. Following stepdown he began passing loose stool 2–3 times per day although faecal polymerase chain reaction and cultures were negative for typical infective causes of diarrhoea, including *C. diff*. A repeat CT scan the day following stepdown (now 5 days after colonoscopy) showed significant reduction in transverse colon thickening with no intramural gas. He remained on the ward for a further 8 days of treatment with benzylpenicillin, clindamycin, meropenem and vancomycin. The patient was discharged 10 days after stepdown from ICU (2 weeks post colonoscopy and initial admission). At this point he had been apyrexial for 11 days, his right upper quadrant pain had resolved, bowel motions had returned to normal and appetite was increasing.

Interestingly, although blood cultures were taken daily, these as well as his initial culture, remained negative. At outpatient review 2 weeks post-discharge (4 weeks post colonoscopy) he had been gradually improving with bloods normalising and weight returning to normal, as he had lost 6 kg during the admission period. CT at this time revealed ongoing colitis of the hepatic flexure and proximal transverse colon, although this was significantly improved from previous studies, and no further intramural gas was visualised. At review 3 months post-discharge, he denied any ongoing symptoms or complications, and reported to be back to his normal full health.

## Discussion and conclusions

This case describes a previously healthy patient who underwent a routine colonoscopy with polypectomy followed by a septic shock state secondary to likely *Clostridium* infection, resulting in abdominal gas gangrene. With aggressive medical management with IV antibiotics, fluids and inotropic support, the patient survived with full recovery at 4 months follow up. Iatrogenic *Clostridium* infection is a rare but documented complication after colonoscopy. In the limited literature surrounding this condition, it has been almost universally fatal, even with aggressive surgical management [7–9].

Colonic gas gangrene is a highly fatal infection, with *Clostridium* toxin production leading to rapid haemodynamic collapse and a rapidly expanding margin of bowel necrosis that can move up to 2 cm per hour [4]. It is caused by infection with Gram positive *Clostridium* species, either through traumatic or spontaneous routes. Traumatic *Clostridium* infections are typically caused by *C. perfringens*, whilst spontaneous infection is typically caused by *C. septicum* and usually occurs in association with gastrointestinal malignancy or immunosuppression [4–6, 10]. *C. perfringens* is a Gram positive, anaerobic, spore forming bacilli, found in soil and normal gastrointestinal flora in 70% of healthy individuals [7]. Systemic signs of gas gangrene infection include shock, septic myocardial depression and haemolytic anaemia [11, 12]. Septic shock in *C. perfringens* infection is mediated by alpha and theta toxins. The exact mechanisms of theta toxin are poorly understood, but it appears to cause a reduction in peripheral vascular resistance, leading to systemic hypotension [13]. Alpha toxin exerts its main effect on the heart, resulting in bradycardia and myocardial depression, while also increasing vascular permeability and inducing haemolysis [11, 12]. This reduction in cardiac output occurs prior to the onset of hypotension, and renders the body unable to compensate for the effect of theta toxin of peripheral vascular resistance, leading to profound shock [12].

One of the hallmarks of necrotizing clostridial infections is the production of gas in tissues. This was evidenced in this case by the presence of pneumatosis intestinalis, the radiographic sign of intramural gas in the gastrointestinal tract. Whilst there are several causes for this appearance (including ischemia, necrotising enterocolitis or gas gangrene infection) this patient’s history, presentation with septic shock and septic myocardial depression and new intravascular haemolysis suggest gas gangrene to be the most likely cause in this case [14, 15].

Three cases of gas gangrene post-colonoscopy have been reported in the literature to date [7–9]. All three of these cases were also associated with polypectomy, as was seen in our case report, however these cases reported retroperitoneal rather than intramural infection [7–9]. These patients were also typically older, ranging from 58 to 61 years of age. The presence of risk factors for gas gangrene was variable. Similar to our patient, 2 of these cases reported no prior medical history and no risk factors for spontaneous gas gangrene [5, 7, 9]. One patient suffered Crohn’s disease although the use of immunosuppressive therapy was not reported [8]. Although not classical, the combination of identification of *C. perfringens* in the reported cases and a lack of risk factors for spontaneous infection seem to imply that this clinical scenario of gas gangrene infection post-polypectomy could be considered a traumatic rather than a spontaneous gas gangrene infection.

The most striking difference between the case reported here and those in the literature is that all 3 cases reported prior were fatal within 48 h of the initial colonoscopy. Misdiagnosis likely plays a role in these poor outcomes with all cases initially being diagnosed as iatrogenic colonic perforation, as opposed to infection, thus delaying initiation of antibiotic therapy [7–9]. The case reported by Shaw et al. received imipenem only after exploratory laparotomy. Similarly, the patient reported by Boenicke et al. was started on cefotaxime and metronidazole after a first exploratory laparotomy, with penicillin and meropenem being only added after a second operation. The case by Gioia et al. received no antibiotic treatment prior to death, which occurred in the operating theatre. An autopsy case series of 8 patients with colonic gas gangrene not in association with colonoscopy revealed a similarly poor outcomes, with only 1 case correctly diagnosed prior to patient death [16]. This case series was limited in the reporting what medical or surgical treatment was provided. This dearth of available literature means clinicians are without high quality evidence in managing this condition.

The choice of antibiotic is controversial and limited by available literature. Penicillins have historically been used as the treatment of choice for clostridial infections although more recent evidence has shown better outcomes using clindamycin or tetracycline antibiotics [10]. This is theorised to be due to the inhibition of toxin synthesis resulting in reduced vasodilation, myocardial depression and thus less severe shock [10]. Alongside antibiotic therapy, surgical debridement is also thought to be important in the management of gas gangrene infection, however evidence exists to suggest a non-operative approach may be a viable option in colonic gas gangrene. Morris et al. reported 97 cases of pneumatosis intestinalis (of which gas gangrene is a cause) and found 50% of cases could be managed non-operatively with no increase in mortality compared to surgical management [14]. However, the number of patients with pneumatosis intestinalis due to infection was not reported in this study, making extrapolation to cases of gas gangrene limited [14]. Another potential treatment option is hyperbaric oxygen, however this is not yet considered standard of care and was unavailable at our institution [10].

In this case there were a number of factors that differentiated this case from existing reports and may have contributed to this patient surviving what has previously been reported as a universally fatal complication. Early recognition, early and appropriate antibiotic therapy and good supportive medical management likely contributed to this patient’s favourable outcome. Gas gangrene is often misdiagnosed initially as perforation, a more common cause of rapid deterioration post-procedure, feared by most gastroenterologists. In previously reported studies, the diagnosis of gas gangrene was made posthumously. Because of the rapid progression of this condition, it is important for gastroenterologists to maintain a wide differential in the unwell patient post-colonoscopy and entertain both medical and surgical causes. In reported studies, antibiotic choice was highly variable, in terms of both timing and agent chosen, as reported above. In our case the patient was initiated on a penicillin at the time of admission and was on full appropriate antibiotics within 24 h of colonoscopy. This, plus his ICU support, was likely a determining factor in his good outcome, even without surgical management.

A limitation of this case is that positive cultures for clostridia where unable to be obtained, making this a presumed gas gangrene infection, however clostridium species are notoriously difficult to culture, and thus negative blood cultures does not rule out infection [17]. Culture diagnosis was only achieved on post-mortem specimens in the three previously described cases, and seven of the eight patients from the autopsy case series [7–9]. It is possible the physiological response observed in this cause could result from other causes of bacterial sepsis or that the observed gas could be a mechanical consequence of polypectomy, however the combination of features described make clostridial myonecrosis highly likely. The strength of this case is that it is, to the authors knowledge, the first report of a patient surviving this rare colonoscopy complication, which may have contributed to the difficulty in obtaining samples for culture.

In conclusion, we present a case of post-colonoscopy gas gangrene successfully treated with medical and antibiotic therapy alone, with resolution of symptoms at 4 months follow-up. This case is to our knowledge the first successful treatment of post-colonoscopy gas gangrene. The patient’s satisfactory clinical course was likely due to early diagnosis and initiation of appropriate antibiotic therapy. We show that gas gangrene can be successfully managed without surgical intervention, but high-quality evidence to support this practice is lacking. Endoscopists should be aware of this rare but potentially lethal condition and consider it in the differential of a rapidly deteriorating patient post-colonoscopy.

## Data Availability

Data sharing is not applicable to this article as no datasets were generated or analysed during the current study.

## Abbreviations

ICU: Intensive Care Ward
SpO2: Blood oxygen saturation
CRP: C Reactive Protein
ALP: Alkaline Phosphatase
GGT: Gamma glutamyl transpeptidase
IV: Intravenous
CT: Computed tomography
